# Ultrasound Imaging Properties of Heterologously Synthesized Gas Vesicles from Halophilic Archaeon

**DOI:** 10.3390/nano16010062

**Published:** 2025-12-31

**Authors:** Wenze Ou, Chenxing Liu, Yuanyuan Wang, Qiuxia Fu, Wei Liu, Huan Long, Fei Yan

**Affiliations:** 1 State Key Laboratory of Quantitative Synthetic Biology, Shenzhen Institute of Synthetic Biology, Shenzhen Institutes of Advanced Technology, Chinese Academy of Sciences, Shenzhen 518055, China; 2 University of Chinese Academy of Sciences, Beijing 100049, China; 3 Key Laboratory of Algal Biology, Institute of Hydrobiology, Chinese Academy of Sciences, Wuhan 430072, China

**Keywords:** gas vesicles, ultrasound imaging, *Haloferax volcanii*, heterologous expression, genetic engineering

## Abstract

Biosynthetic gas vesicles (GVs), as novel nanoscale ultrasound contrast agents, exhibit unique potential in biomedical ultrasound imaging. For example, they are expected to have better tissue penetration through the tumor vasculature for detecting tumor cells by the design of GV-based acoustic probes. Of all these GVs, GVs from *Halobacterium* sp. NRC-1 possess the largest size (over 200 nm) and are nearly spherical in shape, endowing them with stronger acoustic signals and better tumor penetration. However, their genetic manipulation is relatively difficult due to the requirement of a high-salt cytoplasmic environment for their expression and assembly, limiting the application of biosynthetic technology for modulating their structural features in heterologous host cells. In this study, we cloned the gene cluster encoding GVs from *Halobacterium* sp. NRC-1 and transformed it into *Haloferax volcanii*, an archaeal species naturally incapable of producing GVs. The genetically engineered *Haloferax volcanii* successfully synthesized functional GVs (GV_vol_) with a similar size and shape to naturally synthesized GVs from *Halobacterium* sp. NRC-1 (GV_halo_). The ultrasound imaging properties of GV_vol_ heterologously synthesized in *Haloferax volcanii* were compared with naturally synthesized GV_halo_ in vitro and in vivo, showing that GV_vol_ could achieve a mean signal intensity of 113.6 ± 0.9 a.u. in vitro and a peak intensity of 121.5 ± 0.8 a.u. in vivo in the kidney, compared with 115.7 ± 0.5 a.u. and 119.0 ± 0.5 a.u. for GV_halo_, respectively. These findings confirm the functional integrity of heterologously synthesized GV_vol_ and its potential for biomedical applications. Our study provides a solid experimental foundation for genetically tailoring *Halobacterium* GV properties to optimize biomedical imaging performance.

## 1. Introduction

Nanoscale ultrasound contrast agents have attracted extensive attention in medical imaging due to their ability to penetrate blood vessels and accumulate within tumor tissues, significantly expanding their potential applications in disease diagnosis and therapy, especially in tumors [1,2,3]. Traditional ultrasound contrast agents are primarily chemically synthesized, presenting limitations such as complex manufacturing processes, poor biocompatibility, limited stability, and challenges in functional modification [4,5]. To address these shortcomings, biosynthetic nanoscale ultrasound contrast agents have emerged as a promising research frontier. Compared with traditional chemically synthesized agents, biosynthetic nanoscale GVs offer superior biocompatibility, enhanced stability, and the capacity for genetic-engineering-based modification. For example, targeted GVs can be obtained by fusing genetically encoded ligand genes to the GvpC gene, which encodes GV surface protein [6,7]. These features not only enable precise and high-quality imaging, but also facilitate customized synthesis and functionalization, opening new avenues for personalized and accurate tumor diagnosis [8,9,10,11].

Various cyanobacteria and archaea naturally synthesize gas vesicles, which are gas-filled proteinaceous nanostructures that provide buoyancy [9,12]. Cyanobacterial GVs typically exhibit a cylindrical shape with conical ends, whereas archaeal GVs are spindle-shaped. Their dimensions typically range from 100 to 150 nanometers in length and 80 to 250 nanometers in width [9,13]. Compared with cyanobacterial gas vesicles, archaeal gas vesicles generally have a larger particle size and can produce stronger contrast signals. Moreover, their buoyancy allows for facile purification via simple flotation methods [8,14,15]. Among these, the GV operon from the halophilic archaeon *Halobacterium* sp. NRC-1 has been extensively characterized [14,16,17]. However, *Halobacterium* sp. NRC-1 exhibits several limitations, including slow growth rates, high costs associated with high salt usage, instability in transformation systems, and the disability of genetic modifications [14,18,19]. In contrast, *Haloferax volcanii* is a moderately halophilic archaeon that is easy to cultivate [19,20], featuring well-established molecular genetic tools, efficient heterologous gene expression systems, a shorter growth cycle, the absence of endogenous GV synthesis, and the ability to be mass produced with moderate salt supplementation [21,22]. Its relatively fragile cell wall facilitates efficient lysis in aqueous solutions, thereby reducing purification costs for GVs [23,24,25]. Collectively, these attributes make *Haloferax volcanii* an ideal chassis organism for biosynthesis and large-scale production of GVs.

To date, GVs extracted from *Halobacterium* sp. NRC-1 have demonstrated promising ultrasound contrast imaging capabilities in mouse liver and tumor models using clinical ultrasound equipment [13,25,26]. However, naturally synthesized GVs from *Halobacterium* sp. NRC-1 are difficult to genetically modify, which greatly limits their biomedical application. In this study, we cloned the GV gene cluster from *Halobacterium* sp. NRC-1 into the pWL102 shuttle vector and transformed it into *Haloferax volcanii* to achieve heterologous expression of GVs. We systematically compared the physicochemical properties and ultrasound imaging performance of naturally synthesized GVs from *Halobacterium* sp. NRC-1 with heterologously synthesized GVs from *Haloferax volcanii* through in vitro and in vivo experiments. These results provide a theoretical foundation and technical support for large-scale biosynthesis of ultrasound nanoscale GVs using *Haloferax volcanii* as a chassis cell.

## 2. Materials and Methods

### 2.1. Materials

The *Haloferax volcanii* used in this study was purchased from Guangzhou Mingzhou Biotechnology Co., Ltd., Guangzhou, China. The *Halobacterium* sp. NRC-1 and *Escherichia coli JM110* were strains preserved in our laboratory. LB glucose-free medium (containing 10 g of tryptone, 5 g of yeast extract, and 5 g of NaCl per liter) and LB glucose-free agar (LB medium supplemented with 15 g/L agar) for *E. coli* cultivation were obtained from Huankai Microbial (Cat. No. 28324, Guangzhou, China). Bacteriological agar (Cat. No. A620014) was purchased from Sangon Biotech (Shanghai) Co., Ltd., Shanghai, China. C57BL/6 mice were purchased from the Guangdong Provincial Experimental Animal Center (Guangzhou, China).

### 2.2. Construction of GVs—Synthetic Plasmids and Transformation into Haloferax volcanii

*Halobacterium* sp. NRC-1 was inoculated into liquid medium containing 4.2 M NaCl and cultured at 37 °C with shaking at 220 rpm until the mid-to-late logarithmic phase. Genomic DNA from this strain was used as a template for PCR amplification of the target fragment using a high-fidelity DNA polymerase (Phanta Flash Super-Fidelity DNA Polymerase, Vazyme #P521). Primer sequence information: Vac-R: ctattgctcgtgcatttgacgcaccg; Vac-F: tattctcacgcgacacgacctcgaa. The amplified product was directionally cloned into the shuttle vector pWL102 [27]. Positive clones, confirmed by PCR screening, were transformed into the methylation-deficient *Escherichia coli* strain JM110 to eliminate potential Dam/Dcm methylation modifications [28]. Plasmids were extracted and purified using the alkaline lysis method and subsequently introduced into *Haloferax volcanii* via PEG/EDTA-mediated transformation [29] to complete the genetic transformation.

### 2.3. Purification of GVs

For the purification of GV_halo_, *Halobacterium* sp. NRC-1 bacteria were grown at 37 °C on a shaking incubator at 220 rpm/min for 14 days. These bacteria were placed in a separatory funnel for more than 2 weeks to collect the uppermost floated bacteria. Then, these bacteria were lysed by using TMC lysis buffer, and GV_halo_ was isolated through centrifugation at 300 *g* for 4 h. The isolated GV_halo_ was washed with phosphate-buffered saline (PBS) and further purified by centrifugation 3 to 4 times at 300 *g* for 4 h [25]. Finally, the GVs were stored in PBS at 4 C. The concentration of GVs was estimated using a microplate reader (Multiscan GO, Thermo Scientific, Waltham, MA, USA) at a wavelength of 500 nm. For the purification of GV_vol_, *Haloferax volcanii* transformed using the GV plasmid was inoculated in YPC medium containing 4.2 M NaCl and cultured at 42 °C with shaking at 220 rpm for 10 days. After cultivation, the culture was allowed to stand for 1–2 weeks, and the upper cell layer was collected. An equal volume of TMC buffer was added, followed by incubation at room temperature for 24 h to induce cell lysis. The lysate was centrifuged at 250 *g* and 4 °C for 4 h to remove cell debris, and the supernatant was collected. A 200 μL aliquot of the supernatant was mixed with 800 μL of PBS and further purified by centrifugation at 250 *g* and 4 °C for 3 h. The above steps were repeated three times to obtain highly purified GV_vol_, which was stored at 4 °C for subsequent use. The OD_500_ = 3.0 corresponds to 1.0 × 10^12^ particles mL^−1^, with the estimated gas volume being 8.3 μg mL^−1^ of gas.

### 2.4. Characterization of GVs

GV_halo_ and GV_vol_ were diluted 50-fold in PBS and carefully placed upon copper mesh. Then, these specimens were negatively stained using 2% phosphotungstic acid and subsequently left to dry at room temperature for nearly 3 min. The morphologies of GVs were examined by a Transmission Electron Microscope (Hitachi 7700, Hitachi High-Tech, Tokyo, Japan). The particle size and zeta potential were simultaneously measured using dynamic light scattering (DLS) and laser Doppler microelectrophoresis techniques on a Zetasizer Nano S90 instrument (Malvern Panalytical, Malvern, UK). Three independent tubes were prepared for each sample, with each tube measured in triplicate. The results are shown as the mean ± standard deviation (mean ± SD) to ensure the statistical reliability of the data.

### 2.5. In Vitro Ultrasound Imaging

The in vitro ultrasound contrast performance of GV_halo_ and GV_vol_ was assessed at various concentrations using a clinical ultrasound diagnostic system (Mindray Resona R9T, Mindray, Shenzhen, China). The in vitro experiments were conducted at room temperature, which ensures no structural degradation of GVs. Agar phantoms were prepared by dissolving agar powder in distilled water under heat until the solution turned clear and then poured into molds with inserted eppendorf tubes. After cooling and setting, the eppendorf tubes were removed to create agar wells. GVs of different concentrations were added to the wells, and imaging was conducted using an ultrasound device with a linear array transducer (Mindray Resona 9T, Mindray, Shenzhen, China). The parameters were kept as follows: frequency: 7.1 MHz, depth: 2.5, frame rate: 10, dynamic range: 115, contrast gain: 70 dB. To evaluate the impact of MI on GV imaging, the imaging performance of GVs was assessed at different MIs, ranging from 0.095 to 0.281, using GVs at OD500 = 3.0. Based on the selected optimal imaging MI (0.131), further evaluation of the influence of concentration on GV imaging was conducted with an OD500 concentration range of 0.5 to 3.0. Three consecutive frames were continuously acquired for each set of parameters.

### 2.6. In Vivo Ultrasound Imaging

Animal experiments were conducted in accordance with protocols approved by the Ethics Committee of Shenzhen Institutes of Advanced Technology, Chinese Academy of Sciences. For the liver imaging of GVs, male C57BL/6 mice were maintained under isoflurane anesthesia on a 37 °C heating pad. After GV_halo_ and GV_vol_ with OD500 values of 3.0 were intravenously injected into the mice, contrast-enhanced ultrasound was performed using a 7 MHz linear probe driven by a Mindray Resona 9T scanner. The MI was adjusted to the optimal imaging condition for each injection, and the other parameters were set as follows: frequency: 7.1 MHz, depth: 2.5, frame rate: 10, dynamic range: 115, contrast gain: 70 dB. A volume of 100 μL of GVs was administered each time, and imaging was continuously performed for 10 min. For tumor imaging of GVs, C57BL/6 male mice were subcutaneously injected with 1.5 × 10^5^ B16-F10 cells in 100 μL PBS. The B16-F10 line was chosen according to our previous finding that the B16-F10-based tumor model showed robust and comparable GV signals. When the tumor diameter reached over 5 mm, 100 μL of GVs with an OD500 of 3.0 was intravenously injected, and imaging was conducted using the same equipment. Images were continuously acquired for 16 min, and short high-power pulses were applied to burst GVs inside the tumor to prevent interference with the residual ultrasound contrast signals. A wait time of at least 24 h was allowed between two imaging sessions. Image processing and quantification were performed by using ImageJ software (ImageJ/Fiji w11), and the time–intensity curve (TIC) was generated based on the average gray signal value at each time point.

### 2.7. Toxicity Assay

To systematically compare the biocompatibility of gas vesicles derived from distinct bacterial strains, GV_halo_ and GV_vol_ were evaluated. Healthy male C57BL/6 mice were randomly allocated into three groups: a PBS vehicle control, a GV_halo_ group, and a GV_vol_ group. Each animal received a single intravenous dose of 100 μL via the tail vein. Twenty-four hours after injection, whole-blood samples were collected for routine hematological analysis, and major organs (heart, liver, spleen, lung, and kidney) were harvested for paraffin embedding and hematoxylin–eosin (H&E) staining.

### 2.8. Statistical Analysis

The acquired ultrasound images were analyzed using ImageJ software (Fiji). The mean pixel intensity within the region of interest (ROI) placed over each sample was measured and recorded as the ultrasound signal intensity. This specific analysis (quantification of mean pixel intensity) was applied to all in vitro and in vivo image data to generate the signal intensity values and time–intensity curves presented in the figures. The data are expressed as mean ± standard deviation. Independent *t*-tests were used for comparisons between two groups. One-way analysis of variance (ANOVA) followed by a Bonferroni multiple comparison test was used for comparisons among multiple groups. GraphPad Prism software (GraphPad Prism 9) was used for data visualization and statistical analysis. * *p* < 0.05, ** *p* < 0.01, *** *p* < 0.005, and **** *p* < 0.001 were considered statistically significant.

## 3. Results

### 3.1. Heterologous Synthesis of GV_vol_ and Comparison of Physicochemical Properties with GV_halo_

**Figure 1:**
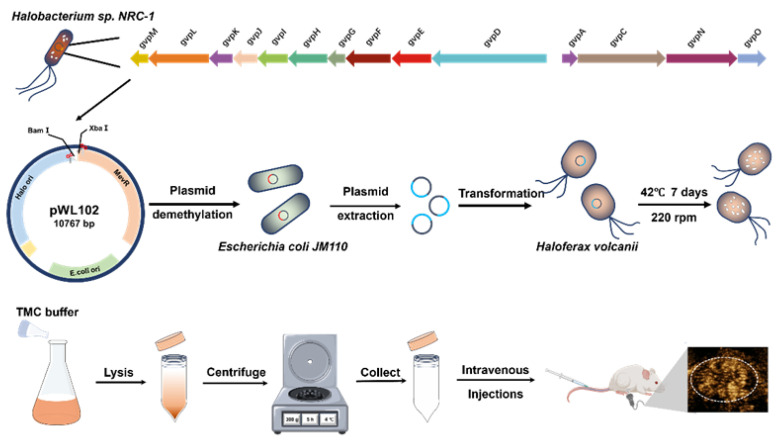
Schematic diagram of the heterologous synthesis of GV_vol_. The 14 genes of the gas vesicle gene cluster (gvpA, gvpB, gvpC, gvpD, gvpE, gvpF, gvpG, gvpH, gvpI, gvpJ, gvpK, gvpL) were cloned from the genome of *Halobacterium* sp. NRC-1 and inserted into the plasmid pWL102 via restriction enzyme digestion and ligation (using BamH I and Xba I sites). The recombinant plasmid was transformed into *Escherichia coli JM110* for demethylation treatment, followed by plasmid extraction. The GV-synthesis plasmid was then transformed into *Haloferax volcanii*, and positive clones were selected. *Haloferax volcanii* harboring the GV-synthesis plasmid was cultured at 42 °C for 7 days to produce heterologously synthesized GV_vol_. The GV_vol_ was released by lysing *Haloferax volcanii* with TMC solution, purified by low-speed centrifugation, and subsequently used for characterization and ultrasound imaging experiments.

**Figure 2:**
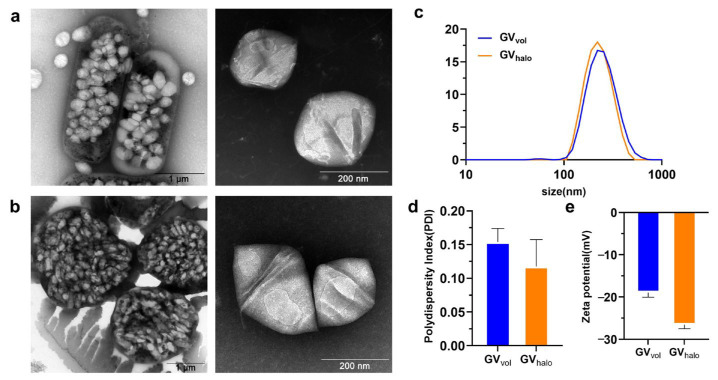
Characterization of GV_halo_ and GV_vol_. (**a**) TEM image of GV_halo_ naturally synthesized by *Halobacterium* sp. NRC-1. (**b**) TEM image of GV_vol_ heterologously synthesized by *Haloferax volcanii*. (**c**) Particle size distribution of GV_halo_ and GV_vol_. (**d**) Polydispersity index (PDI) of GV_halo_ and GV_vol_. (**e**) ζ-potential of GV_halo_ and GV_vol_.

The GV biosynthetic gene cluster was amplified from the DNA of *Halobacterium* sp. NRC-1 and then cloned into the pWL102 shuttle vector, which was then transformed into *Haloferax volcanii* for heterologous expression (Figure 1). GVs from *Halobacterium* sp. NRC-1 (GV_halo_) and heterologously expressed GVs from *Haloferax volcanii* (GV_vol_) were extracted and purified following previously reported protocols [13,25]. Negative-staining TEM revealed that both GV_halo_ and GV_vol_ exhibited similar biconical morphologies (Figure 2a,b). Dynamic light scattering (Zetasizer) measurements showed that GV_halo_ had an average diameter of 226.1 ± 3.3 nm, while GV_vol_ averaged 229.8 ± 2.4 nm (Figure 2c). Their polydispersity indices (PDIs) were 0.118 ± 0.044 and 0.153 ± 0.020, respectively (Figure 2d), indicating similar average sizes with relatively narrow distributions. In terms of surface charge, GV_halo_ displayed a ζ-potential of −26.57 ± 1.06 mV, whereas GV_vol_ showed a slightly lower absolute value of −19.10 ± 1.82 mV (Figure 2e). The −19 mV ζ-potential of GV_vol_ is higher than commercial microbubbles (Sonovue ≈ −31 mV, Sonazoid ≈ −39 mV), indicating that GV_vol_ has less electrostatic interaction with vascular walls [30]. Overall, GV_vol_ closely resembled GV_halo_ in particle size, dispersity, and surface charge, suggesting that it may possess comparable potential for ultrasound imaging applications.

### 3.2. In Vitro Ultrasound Imaging of GVs

**Figure 3:**
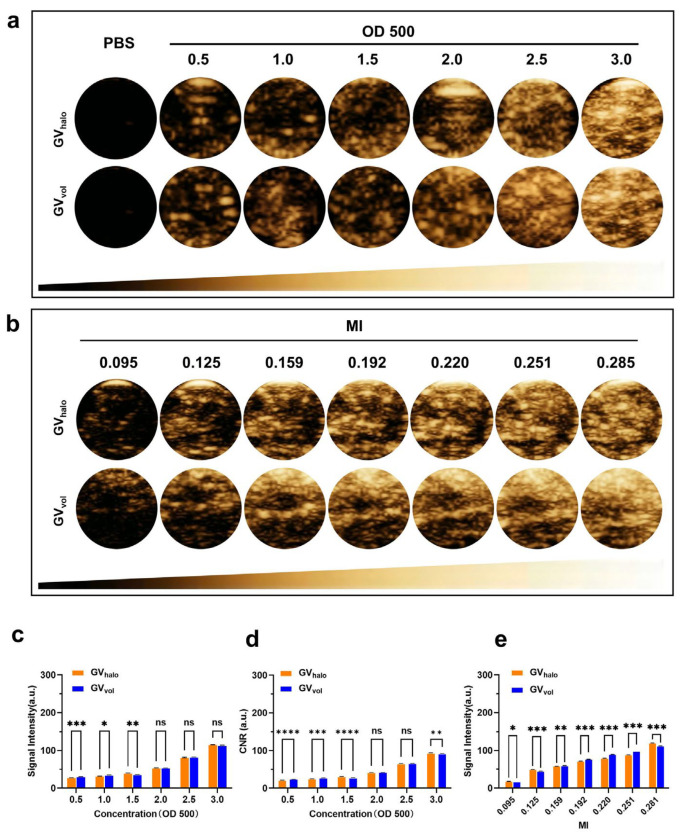
Ultrasound contrast imaging of GVs in vitro. (**a**) Nonlinear contrast images of GVs at different concentrations (OD_500_ = 0.5–3.0). (**b**) Nonlinear contrast images of GVs under different mechanical indices (MI = 0.095–0.285). (**c**) Quantification of the contrast signal intensity of gas vesicles at different concentrations. (**d**) Quantification of the normalized contrast signal intensity (per OD_500_ unit) across OD_500_ concentrations. (**e**) Quantification of the contrast signal intensity of gas vesicles under different mechanical indices. Statistical significance levels are denoted as * for *p* < 0.05, ** for *p* < 0.01, *** for *p* < 0.005, **** for *p* < 0.001 and ns for no statistical significance.

Previous studies have demonstrated that GV_halo_ exhibits remarkable ultrasound contrast enhancement capabilities [13,25]. To evaluate the ultrasound imaging performance of GV_vol_ in agar-based phantom models, we conducted a parallel comparison of ultrasound imaging signals between GV_halo_ and GV_vol_ using a clinical ultrasound system (Mindray Resona R9T). We first examined the effect of GV concentration on imaging signals. As the GV concentration increased from OD_500_ 0.5 to 3.0, both GV_halo_ and GV_vol_ exhibited a dose-dependent enhancement in signal intensity (Figure 3a,c). Specifically, at OD_500_ = 3.0, the mean signal intensity of GV_halo_ reached 115.7 ± 0.5 a.u., comparable to that of GV_vol_ (113.6 ± 0.9 a.u.) (Figure 3a,c). To evaluate contrast efficiency, the CNR was calculated as (I_GV_ − I_background_)/σ. The CNR linearly increased with GV concentration for both GV_halo_ and GV_vol_ (Figure 3d), reaching 92.9 ± 0.4 per OD_500_ for GV_halo_ and 91.1 ± 0.6 per OD_500_ for GV_vol_ at OD_500_ = 3.0. No significant difference was observed between the two agents, indicating that they had comparable acoustic imaging performance under the same OD value. We then investigated the effect of mechanical index (MI) values on imaging performance. Results showed that signal intensity increased with MI for both GVs. At MI = 0.281, GV_halo_ achieved a mean signal intensity of 120.3 ± 0.2 a.u., whereas GV_vol_ reached 112.0 ± 0.3 a.u. (Figure 3b,e). In summary, although GV_vol_ exhibited slightly lower mean signal intensities than GV_halo_ in vitro, both demonstrated robust contrast enhancement performance. These findings indicate that GV_vol_, heterologously synthesized in *Haloferax volcanii,* possesses favorable in vitro ultrasound imaging capabilities, underscoring its potential as a nanoscale ultrasound contrast agent.

### 3.3. In Vivo Ultrasound Imaging of GVs

**Figure 4:**
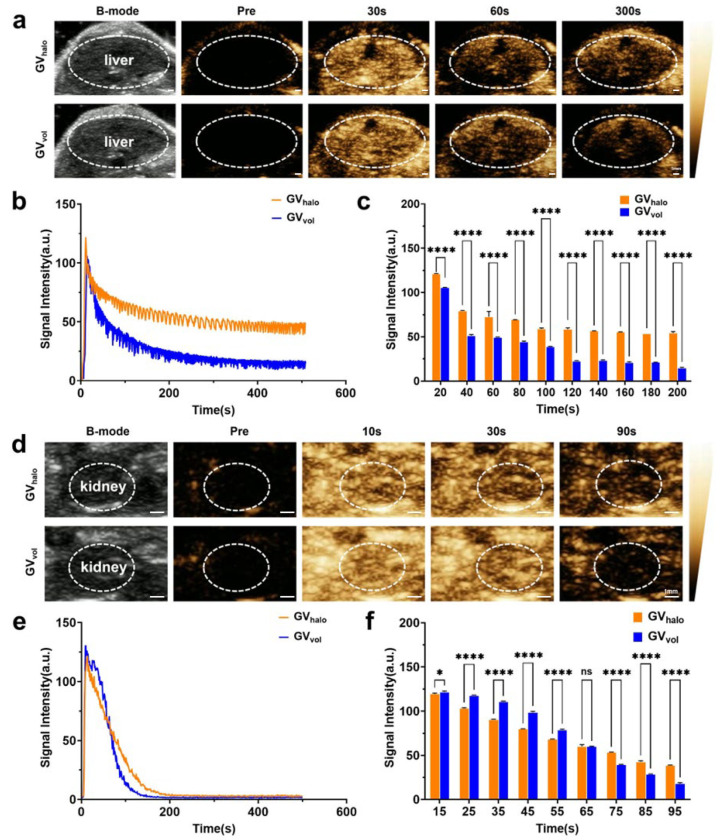
Ultrasound contrast imaging of GVs in vivo. (**a**) B-mode and contrast images of the liver at different time points after tail vein injection of GV_halo_ and GV_vol_ at OD500 = 3.0, scale bar: 1 mm. (**b**) Signal intensity–time curves of GV_halo_ and GV_vol_ in the liver over 5 min. (**c**) Contrast signal intensity of GVs in the liver at different time points. (**d**) B-mode and contrast images of the kidney at different time points after tail vein injection of GV_halo_ and GV_vol_ at OD500 = 3.0, scale bar: 1 mm. (**e**) Signal intensity–time curves of GV_halo_ and GV_vol_ in the kidney over 5 min. (**f**) Contrast signal intensity of GVs in the kidney at different time points. Statistical significance levels are denoted as * for *p* < 0.05, **** for *p* < 0.001, and ns for no statistical significance.

To further evaluate the in vivo imaging capabilities of the two types of GVs, GV_halo_ and GV_vol_ were intravenously injected into C57BL/6 mice via the tail vein, followed by ultrasound imaging in both B-mode and contrast mode. For liver imaging, the signal intensity of GVs in the liver increased over time, reaching a peak at 20 s post-injection, with GV_halo_ and GV_vol_ achieving peak intensities of 121.0 ± 0.7 a.u. and 105.5 ± 0.5 a.u., respectively. Thereafter, the signal gradually declined but remained at relatively high levels within 300 s (Figure 4a–c). For kidney imaging, contrast-enhanced signals of both GVs also increased over time, peaking at 15 s post-injection, with GV_halo_ and GV_vol_ reaching peak intensities of 119.0 ± 0.5 a.u. and 121.5 ± 0.8 a.u., respectively. The elevated signal intensity was maintained for up to 90 s (Figure 4d–f). Overall, the in vivo imaging results indicate that, although GV_vol_ exhibited slightly lower signal intensity than GV_halo_ in the liver, both GVs rapidly and sustainably enhanced ultrasound contrast signals in the liver and kidney after injection, demonstrating the feasibility and application potential of heterologously synthesized GV_vol_ for in vivo ultrasound imaging.

### 3.4. Ultrasound Burst Experiment of GVs

**Figure 5:**
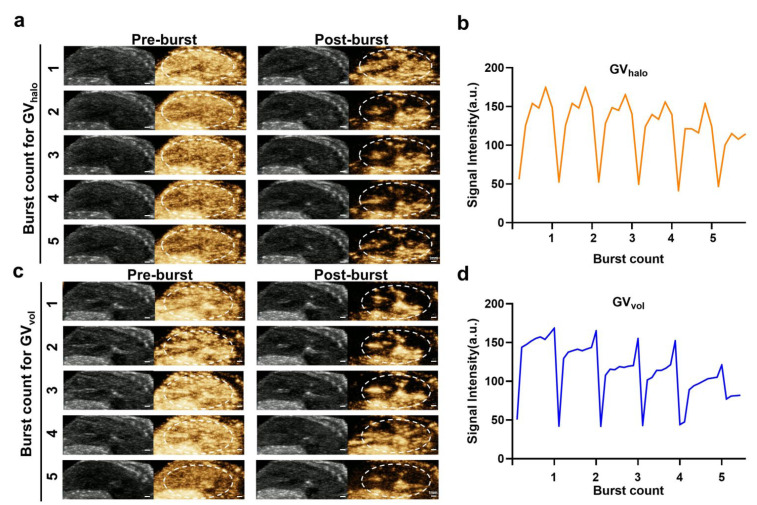
Reperfusion of GV_halo_ and GV_vol_ after multiple ultrasonic bursts in vivo. (**a**) B-mode and contrast images of GV_halo_ during hepatic reperfusion after five ultrasonic bursts, scale bar: 1 mm. (**b**) Time–intensity curve of contrast signals of GV_halo_ during five ultrasonic bursts in the liver. (**c**) B-mode and contrast images of GV_vol_ during hepatic reperfusion after five ultrasonic bursts, scale bar: 1 mm. (**d**) Time–intensity curve of contrast signals of GV_vol_ during five ultrasonic bursts in the liver.

To further verify that the imaging signals were generated by GVs, we intravenously injected both GVs into C57BL/6 mice via the tail vein, followed by the application of short, high-power ultrasound pulses (MI: 0.581; AP: 50.12%) to disrupt the GVs. Liver imaging was monitored in real time during the process. The results showed that, immediately after exposure to the high-energy pulses, the imaging signals from both GV_halo_ and GV_vol_ dropped sharply, but gradually reappeared in the liver thereafter (Figure 5a–d). This disappearance–reappearance pattern of contrast enhancement was consistently observed across five repeated high-power pulse applications. These findings not only confirm that the contrast signals originate from GVs, but also demonstrate that both GV_halo_ and GV_vol_ can be disrupted by ultrasound pulses.

### 3.5. Imaging of Tumors by GVs

**Figure 6:**
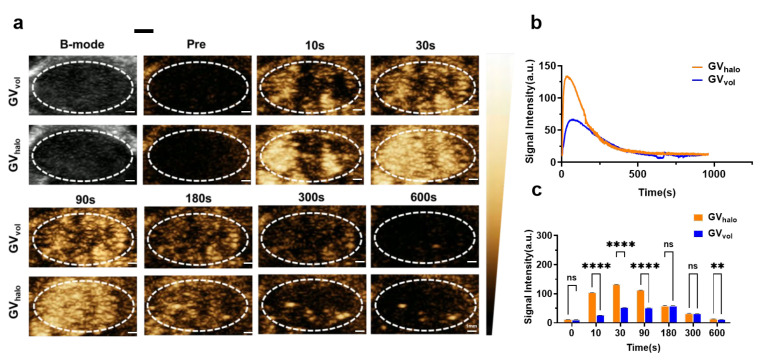
Ultrasound imaging of GV_halo_ and GV_vol_ in tumors. (**a**) Nonlinear contrast images of tumors at different time points after tail vein injection of GV_halo_ and GV_vol_ at OD_500_ = 3.0, scale bar: 1 mm. (**b**) Signal intensity–time curves of GV_halo_ and GV_vol_ within tumors over 15 min. (**c**) Contrast signal intensities of GV_halo_ and GV_vol_ in tumors at different time points. Statistical significance levels are denoted as ** for *p* < 0.01, **** for *p* < 0.001, and ns for no statistical significance.

After confirming the excellent imaging performance of GV_halo_ and GV_vol_ in the liver and kidneys, we further investigated their contrast enhancement capabilities in tumor imaging. GV_halo_ and GV_vol_ with an OD500 of 3.0 were intravenously injected via the tail vein into mice bearing B16-F10 tumors. The results showed that both GV_halo_ and GV_vol_ effectively accumulated within tumor tissues (Figure 6a–c). GV_halo_ and GV_vol_ reached their respective peak signal intensities at 30 s and 90 s post-injection, followed by a gradual decline. Specifically, the peak signal intensity of GV_halo_ was 133.26 ± 0.371 a.u., whereas that of GV_vol_ was 62.81 ± 0.33 a.u. Moreover, the imaging signals generated by both types of GVs were sustained for approximately 300 s. Taken together, these findings suggest that, although GV_vol_ exhibited a substantially lower peak signal intensity in tumors than GV_halo_, it still holds considerable potential for tumor imaging, particularly in scenarios where extended imaging windows and stable signal maintenance are critical for diagnostic accuracy or longitudinal monitoring.

### 3.6. Biosafety of GVs

**Figure 7:**
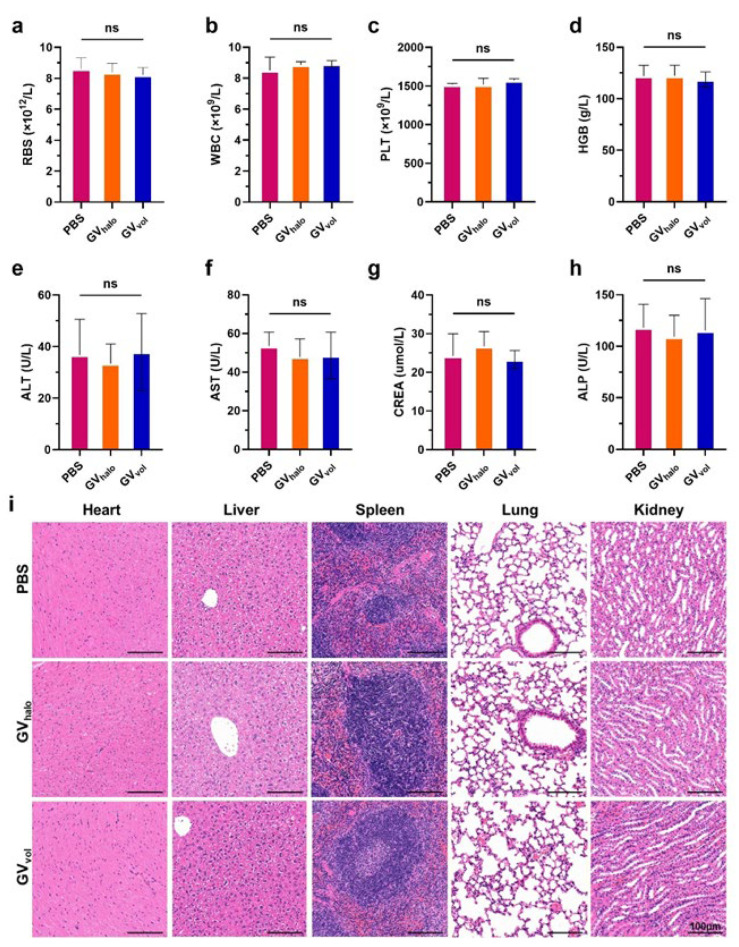
Biosafety of GV_halo_ and GV_vol_. (**a**–**h**) Serum biochemical parameters measured at 24 h post-injection of PBS or gas vesicles, including RBC, WBC, PLT, HGB, ALT, AST, BUN, and CREA. Statistical significance levels are denoted as ns for no statistical significance. (**i**) Representative H&E-stained sections of heart, liver, spleen, lung, and kidney collected from mice treated with GV_halo_, GV_vol_, or PBS, scale bar: 100 μm.

To assess the in vivo biosafety of distinct GV phenotypes, we evaluated GV_halo_ and GV_vol_. Following systemic administration in mice, both GV types exhibited favorable biocompatibility profiles. Hematological indices, including RBC, WBC, PLT, and HGB, remained within normal ranges, as did hepatic (ALT, AST) and renal (BUN, CREA) function markers (Figure 7a–h). Histological analysis via H&E staining revealed no observable pathological changes in major organs such as the heart, liver, spleen, lung, and kidney (Figure 7i). These 24 h results indicate that GV_halo_ and GV_vol_ do not induce acute systemic toxicity or tissue damage within this short-term observation window, supporting their initial biocompatibility and short-term safety profile for applications such as ultrasound contrast imaging.

## 4. Discussion

In recent years, the development of various nanoscale contrast agents has brought about a revolutionary advance in the field of ultrasound contrast imaging. In particular, GVs biosynthesized by cyanobacteria and archaea have emerged as potential alternatives to traditional chemically synthesized contrast agents [31]. However, the natural producers of GVs, such as cyanobacteria and archaea, have notable drawbacks, including long cultivation cycles, difficulties with large-scale culture, and limitations imposed by naturally synthesized GVs that hinder the customization of GVs with different properties via synthetic biology approaches. Therefore, it is critical to identify a chassis organism that cannot naturally synthesize GVs but has a short growth cycle, low cultivation and purification costs, and a well-established genetic engineering and synthetic biology toolbox. Such a chassis would be a key step toward large-scale biosynthesis of nanoscale ultrasound contrast agents.

In this study, we successfully achieved the heterologous expression of the GV gene cluster from *Halobacterium* sp. NRC-1 in the halophilic archaeon *Haloferax volcanii* and systematically compared the physicochemical properties and ultrasound imaging performance of heterologously synthesized GV_vol_ with naturally synthesized GV_halo_. The results demonstrated that GV_vol_ was highly similar to GV_halo_ in morphology, particle size, dispersity, and surface charge, confirming the feasibility of *Haloferax volcanii* as a functional GV biosynthesis chassis. In vitro imaging experiments further revealed that GV_vol_ generated strong ultrasound contrast enhancement signals in a concentration- and mechanical-index-dependent manner, with performance comparable to GV_halo_, highlighting its potential as a nanoscale ultrasound contrast agent. Notably, the acoustic performance of analogous GVs has shown to be comparable to clinically used ultrasound contrast agents such as Sonovue and Sonazoid in terms of second-harmonic signal intensity, supporting the translation potential of GVs.

In vivo experiments showed that GV_vol_ could rapidly and sustainably enhance ultrasound signals in the liver and kidney, although its peak intensity was slightly lower than that of GV_halo_. Notably, both types of GVs exhibited the reversible disappearance and reappearance of contrast signals under high-power ultrasound pulses, suggesting their potential for targeted drug delivery via ultrasound-triggered destruction. Furthermore, tumor imaging experiments demonstrated that GV_vol_ could effectively penetrate tumor tissue and maintain persistent contrast signals, primarily due to the enhanced permeability and retention (EPR) effect [32,33,34]. Although the peak tumor signal of GV_vol_ was lower than that of GV_halo_, this indicates that GV_vol_ is suitable for applications requiring prolonged imaging windows or stable signals, offering potential for tumor-targeted imaging and theranostic integration.

The multifunctionality and high tunability of genetically engineered GVs hold great promise for diverse applications, including molecular imaging, ultrasound-mediated drug delivery, in vivo cell tracking, and acoustic manipulation [35,36]. The biomedical application of GVs can not only expand their value in disease diagnosis and treatment but also deepen our understanding of the mechanisms underlying cell therapies [37,38]. Overall, this study demonstrates the great potential of genetically engineered GVs as novel ultrasound contrast agents and validates the feasibility of *Haloferax volcanii* as a scalable, genetically tractable GV production platform. GV_vol_ successfully recapitulated the key properties of GV_halo_, laying the foundation for large-scale biosynthesis of functional nanoscale ultrasound contrast agents and providing a versatile tool for molecular imaging, tumor diagnosis, and potential ultrasound-mediated therapies. While our 24 h data support acute biocompatibility, future studies are still needed to assess long-term safety and immune responses.

Despite these promising results, our study still has some limitations. The biosafety assessment is preliminary, and the in vivo pharmacokinetics were not defined. Furthermore, the subtle differences in the signal intensity between GV_vol_ and GV_halo_, probably attributable to the variations in GV formation or organ-specific uptake due to their distinct biosynthetic origins, still need further mechanistic investigation.

Future research needs to explore the in vivo clearance pathways and biodistribution of GVs to elucidate their pharmacokinetic profile, determine the optimal conditions for large-scale biosynthesis of GVs using *Haloferax volcanii* as a chassis, enhance the signal intensity, improve the particle size uniformity and dispersity, optimize the in vivo stability, and incorporate functional payloads through genetic modifications. These efforts could enable the combination of imaging and therapeutic applications, thereby advancing the use of biosynthetic GVs in precision medicine.

## Data Availability

Data are contained within the article.
